# Anesthesiological risks in mucopolysaccharidoses

**DOI:** 10.1186/s13052-018-0554-1

**Published:** 2018-11-16

**Authors:** Alessandra Moretto, Maria Grazia Bosatra, Laura Marchesini, Simonetta Tesoro

**Affiliations:** 1 Department of Perioperative Medicine and Intensive Care, ASST San Gerardo, Via Pergolesi 33, 20900 Monza, MB Italy; 2 Department of surgical and biological sciences, Section of Anesthesia, Analgesia and Intensive care, Perugia University, Azienda Ospedaliero Universitaria di Perugia, Piazzale Menghini 1, 06100 Perugia, PG Italy

**Keywords:** Mucopolysaccharidosis, Anesthesia, Perioperative complications, Airway management

## Abstract

**Background:**

Patients suffering from mucopolysaccharidosis are among the most complex from the anesthesiological point of view, especially regarding the management of the airway. The evidence base for anesthesia management is often limited to case reports and small case series.

**Aims:**

To identify useful information about experience with each subtype of mucopolysaccharidosis reported in the literature and propose a guide on the best options for airway management to the anesthesiologists who take care of these patients.

**Methods:**

A query of the PubMed database specific for “anesthesia” and “mucopolysaccharidosis” and a further query specific for “mucopolysaccharidosis and difficult airway management” was conducted. We looked for those items that offered practical guidance to anesthesiological management. We did not exclude case reports, especially those that reported a specific technique, because of their practical suggestions.

**Results:**

We identified 15 reviews, 17 retrospective case series, 5 prospective studies, and 28 case reports that focused on airway managements in anesthesia or had practical suggestions for preoperative evaluation and risk assessment. An accurate preoperative evaluation and the need for an experienced team are emphasized in all the reviewed articles and for each type of mucopolysaccharidosis. Many suggestions on how to plan the perioperative period have been highlighted. Insertion of a laryngeal mask airway generally improves ventilation and facilitates intubation with a fiberoptic bronchoscope. Furthermore, the videolaryngoscope is very useful in making intubation easier and facilitating bronchoscope passage.

**Conclusions:**

Patients with mucopolysaccharidosis are at high risk for anesthesia-related complications and require a high level of attention. However, a multidisciplinary approach, combined with expertise in the use of new techniques and new devices for airway management, makes anesthesiological management safer. Further research with prospective studies would be useful.

**Electronic supplementary material:**

The online version of this article (10.1186/s13052-018-0554-1) contains supplementary material, which is available to authorized users.

## Background

Mucopolysaccharidosis (MPS) involves defective activity of the lysosomal enzymes, which blocks degradation of mucopolysaccharides and leads to abnormal accumulation of heparan sulfate, dermatan sulfate, and keratan sulfate (collectively known as glycosaminoglycans or GAGs).

MPS can be classified as follows: Hurler syndrome (MPS IH), Hurler-Scheie syndrome (MPS IH/S), Scheie syndrome (MPS IS), Hunter syndrome (MPS II), Sanfilippo syndrome (MPS IIIA, B, C, D), Morquio syndrome (MPS IVA, B), Maroteaux-Lamy syndrome (MPS VI), Sly syndrome (MPS VII), and MPS IX. The etiology, treatment options, and common symptoms are detailed in Table 1.

**Table 1:** Mucopolysaccharidoses (MPS) classification

Type	Common name	Major symptoms	Deficient enzyme	GAGs	Enzyme replacement therapy
MPS IH	Hurler syndrome	Progressive involvement of the heart (cardiomyopathy cardiac valve and coronary infiltration), skeleton, and airways. Frequent obstructive sleep apnea. Possible cervical spine involvement. Progressive intellectual disability	α-l-iduronidase	Heparan sulfate Dermatan sulfate	Aldurazyme
MPS IHS	Hurler-Scheie syndrome	Intermediate severity, onset in early childhood with mild to cognitive impairment			
MPS IS	Scheie syndrome	Least severe, onset in childhood with no cognitive impairment			
MPS II	Hunter syndrome	Wide range (mild to severe forms). In severe forms progression similar to MPS IH. Cardiac valve and coronary infiltration cardiomyopathy. Frequent obstructive sleep apnea. Intellectual disability may be absent in mild form	Iduronate sulfate sulfatase	Heparan sulfate Dermatan sulfate	Elaprase
MPS IIIA	Sanfilippo syndrome A	Developmental delay, severe hyperactivity, behavioral problems. Somatic manifestations are generally less severe than other MPS	Heparan-S-sulfaminidase	Heparan sulfate	Not available
MPS IIIB	Sanfilippo syndrome B	Symptoms and disease progression are less severe than IIIA	N-acetyl-α-d-glucosaminidase		
MPS IIIC	Sanfilippo syndrome C		Acetyl-Co-A glucosaminidase		
MPS IIID	Sanfilippo syndrome D		N-Acetylglucosidase N-acyltransferase		
MPS IVA	Morquio syndrome A	Severe skeletal dysplasia usually leading to pulmonary compromise. Hypoplasia of the odontoid process causing atlanto-axial instability and cervical subluxation. Aortic valve involvement common. Usually intellectually normal	Galactosamine-6-sulfate sulfatase	Keratan sulfate Chondroitin 6-sulfate	Elosulfase alfa
MPS IVB	Morquio syndrome B	Milder than MPS IVA			
MPS VI	Maroteaux–Lamy syndrome	Severe skeletal dysplasia, spinal cord compression from GAGs. Progressive cardiac valve degeneration with stenosis and/or incompetence	N-acetyl-galactosamine α-4-sulfate sulfatase	Dermatan sulfate	Galsulfase, Naglazyme
MPS VII	Sly syndrome	Highly variable developmental delay and progressive intellectual disability may be present	β-glucuronidase	Dermatan sulfate, heparan sulfate, chondroitin sulfate	recombinant human β-glucuronidase
MPS IX		Periarticular soft tissue masses, mild short stature, and acetabular erosions without classical MPS features It is very rare	Hyaluronidase 1	Hyaluronan	

*GAG* glycosaminoglycan

Patients with MPS show normal initial development, with abnormalities appearing in infancy or later in childhood. Symptoms are characterized by having a broad spectrum of severity in the expression of the musculoskeletal and neurological manifestations and these may precede the diagnosis. Consequently, many procedures are performed prior to a diagnosis being made [1–3].

Table 2 lists the most common surgeries that are required as a consequence of GAG accumulation as reported by Arn et al. [2, 4].

**Table 2:** Most common surgical procedures in mucopolysaccharidosis type I patients. They are also common in other types of MPS [2, 4]

• Myringotomies and related procedures	
• Adenotonsillectomy	
• Tracheostomy	
• Nasal and sinus procedures	
• Corneal transplant procedures and other eye interventions	
• Cardiac valve replacement and reconstruction	
• Umbilical and inguinal hernia repair	
• Hydrocele, phimosis, repair and other genitourinary procedures	
• Abdominal interventions and feeding tubes	
• Tendon release, carpal tunnel, spinal decompression, hip, knee, foot and other orthopedic surgery	
• Ventriculoperitoneal shunt	
• Tooth extraction or repair and other oral surgery	

In this article, we want to deal specifically with anesthesiological problems; specific topics on MPS (types, prevalence, therapies, neurosurgical, orthopedic, cardiological, respiratory aspects, etc.) are covered in detail in other articles in this Supplement.

**Fig. 1:**
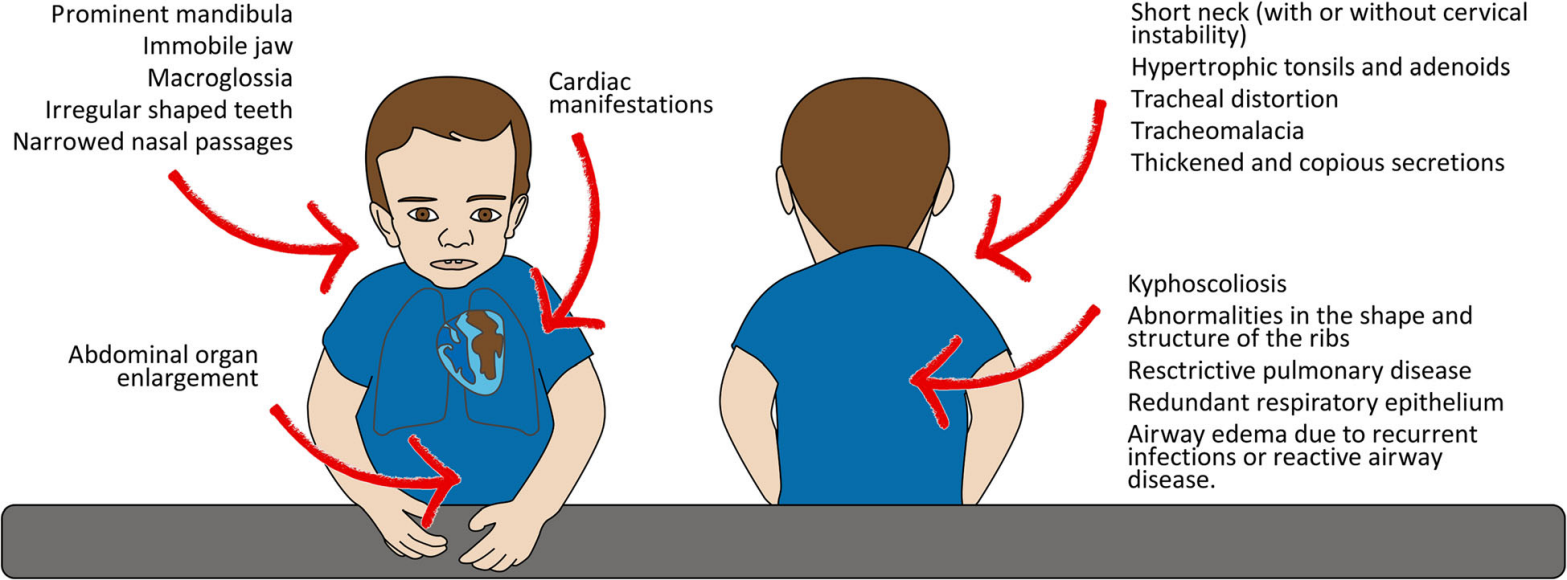
The most common clinical features that increase the anesthesiological risk in MPS patients, with great variability among different type of MPS or among different phenotypical expression within the same type

The most common characteristics that increase the risk in cases of anesthesia are summarized in Fig. 1 and in Table 1. MPS I, II, and VI show very similar aspects; MPS IVA also shares these characteristics, although skeletal involvement is more apparent. Patients with MPS III may also have disease processes which can complicate anesthesiological management, although somatic manifestations are generally less severe [3, 5–7].

The high prevalence of perioperative complications underlines the critical role of a multidisciplinary approach with codified surveillance protocols. The risk of difficult intubation must always be suspected and requires experienced staff expert in algorithms for difficult intubations and in the use of advanced devices for airway management.

## Methods

We reviewed the literature for reports of anesthesiological and airway-related complications in patients with MPS. In March 2017, LM and ST conducted a query of the PubMed database, specifically concerning “anesthesia” and “mucopolysaccharidosis” and a further query specifically concerning “mucopolysaccharidosis and difficult airway management.” Together, all the authors selected articles containing practical information for anesthesiological management of MPS patients and, in case series or larger studies, they identified the various types of MPS contained therein. A PRISMA flow diagram is included as (Additional file 1).

## Results

The review of the literature identified 185 records. Records after duplicates were removed totaled 133, and 34 have been excluded (21 were not in English, 11 were published before 1980, and in two instances the full text was not available). From the 99 full-text articles assessed for eligibility, 34 have been excluded (eight due to description of drugs and devices no longer in use, 22 case reports with no practical suggestions, and four not focused on anesthesiological management). We identified 15 reviews, 17 retrospective case series, five prospective studies (none of which were randomized and only one controlled), and 28 case reports. Among the case reports, two focused on the problem of spinal stability during induction of general anesthesia in children, 12 focused on airway management in anesthesia in children (in most of them the laryngoscopic view and/or technique for fiberoptic bronchoscope (FOB) intubation, for videolaryngoscope intubation, and for laryngeal mask airway (LMA) insertion are described), four focused on loco-regional anesthesia, and 10 focused on adult patients (which contain detailed descriptions of airway management with FOB or with FOB through LMA, information about sedation technique, or reports of serious complications).

### Mucopolysaccharidosis type I

In patients with MPS I, soft tissue storage and the skeletal system are affected with or without brain disease (Table 1). Symptoms such as frequent respiratory and ear infections, chronic nasal discharge, and enlargement of the tongue, tonsils and adenoids are often present, and may precede the diagnosis [8]. MPS I patients are at higher risk of difficult airway management [6, 8–10]. A recent analysis found that about 20% of deaths associated with surgery in patients with MPS I are directly related to airway obstruction or difficult intubations [2]. Approximately 75% of patients enrolled in the MPS I Registry reported at least one surgical procedure in childhood [4]. Retrospective case series studies of anesthesia charts, mostly performed on data predating the enzyme replacement therapy or hematopoietic stem cell transplantation era, showed difficult intubation in more than 40% and failed conventional intubation in more than 12% (mean age around 5 years) [7, 10, 11]. Another study, which included four MPS I patients (mean age 5 years), found a lower incidence of difficult intubation, perhaps because these patients had received the benefit of early treatment [12].

### Mucopolysaccharidosis type II

In patients with MPS II, as in MPS I, soft tissue storage and the skeletal system are affected with or without brain involvement [10] (Table 1). The short neck, immobile jaw, and pathological changes in the upper airways make general anesthesia a difficult and high-risk procedure [6, 7, 10, 12–14]. In mild forms, surgery may sometimes precede diagnosis. Busoni et al. reported a case study of an 11-year-old MPS II boy who had stridor as a clinical sign in which an LMA failed to secure the airway because of a polypoid formation above the epiglottis [15]. In another case report, delayed awakening and postoperative respiratory depression were reported with low fentanyl doses, suggesting an increased sensitivity to anesthetics [16].

### Mucopolysaccharidosis type III

In patients with MPS III, the central nervous system is always affected (Table 1). No or very few difficult intubations have been reported in retrospective studies, even when multiple anesthetic procedures were performed in the same child at different ages [6, 7, 10, 12, 17, 18]. A recent prospective study evaluated the incidence of airway issues and complications with general anesthesia in 25 patients with MPS IIIA and B who underwent magnetic resonance imaging (MRI) and lumbar puncture. Deep sedation with a native airway and spontaneous ventilation was provided without major complications with dexmedetomidine and propofol. Although upper airway obstruction and desaturations were noted, they were resolved with simple airway maneuvers without further airway intervention, and all patients were discharged to home on the same day [5].

### Mucopolysaccharidosis type IVA

In patients with MPS IVA the skeletal system is primarily affected. Multiple abnormalities subject the patient to high anesthetic risk [6, 7, 10, 12, 19–25]. Tomatsu et al. reported a case series where 67% of patients presented tracheal narrowing that worsen with age [26]. Tong et al. describe the case of a 16-year-old MPS IVA patient who developed paraplegia due to thoracic spinal cord infarction during spinal decompression. It is therefore important to establish intraoperative neuromonitoring baseline assessments prior to turning patients to the prone position following induction of anesthesia and to monitor cardiac output during prone positioning [23]. In a retrospective review, only one out of six MPS IVA patients presented poor laryngoscopic visibility, probably due to limited neck mobility caused by a previous cervical fusion [10]. In the study by Frawley et al., the only MPS IVA patient included did not present any anesthetic complications [12]. The largest study on MPS IVA patients is a retrospective and descriptive study of 28 children; eight patients (seven of them with the cervical spine surgically fused) were difficult to intubate. Part of the intubations performed were conventional laryngoscopies using in-line stabilization with neutral head and neck position or with videolaryngoscope as soon as it was available. Four children had perioperative complications, mainly due to cervical spine instability and GAG deposits in the trachea [27].

### Mucopolysaccharidosis type VI

In patients with MPS VI, soft tissue storage and the skeletal system are affected. Risk of paralysis is present whenever an MPS VI patient undergoes any surgical procedure requiring anesthesia. The literature reports some cases or case series where the anesthetic procedure is described. Direct laryngoscopy [28], videolaryngoscope [29], or FOB [30] have been used as intubation techniques. In one case, intraoperative somatosensory evoked potential monitoring detected acute spinal cord compression, probably due to the slightly altered neck position during surgery [30].

### Mucopolysaccharidosis types VII and XI

No specific information about anesthesia is reported on these very rare MPS.

## Discussion

The high anesthetic risk for MPS patients consists primarily in the predicted difficult airway and in the presence of comorbidity. This underlines the critical role of an appropriate anesthesiological plan.

### Pre-operative evaluation

A thorough preoperative evaluation must be carried out using a multidisciplinary approach. The anesthesiologist must examine all the diagnostic tests performed. Ear, nose, and throat (ENT) assessment and the Mallampati classification system (based on the visibility of tonsils, pillars, uvula, and soft palate) can evaluate nostril narrowing, adeno-tonsillar hypertrophy and macroglossia, nasopharyngeal obstruction, and supraglottic narrowing [31]. Whenever surgery is planned, it would be desirable to perform a computed tomography scan (CT) of the airway with an extension of the scan to create a three-dimensional reconstructions of the trachea [32]. Sleep studies conducted overnight during natural sleep can detect obstructive sleep apnea and could suggest the need for postoperative monitoring and therapies in the intensive care unit to maximize respiratory function [8, 13, 14, 31, 33]. Patients may develop airway occlusion upon neck flexion and adopt a “sniff position” to preserve airway patency [31]. Compromised respiratory function due to restrictive disease with decreased lung volumes and ventilation-perfusion mismatching is a possible complication. Chronic hypoxemia over time can have cardiovascular consequences, such as pulmonary hypertension leading to cardiorespiratory failure [8, 13, 14, 19]. The results of pulmonary function tests must be taken into consideration [14, 31, 33]. Examinations or functional assessments and routine spine x-rays, MRI, and flexion-extension cervical film assessments may confirm the potential for atlanto-axial subluxation, which is a contraindication for cervical extension during endotracheal intubation. Early signs of myelopathy could eventually be an indication for prophylactic cervical spine fusion. Intraoperative neuromonitoring with somatosensory evoked potentials is suggested during surgery to detect any acute spinal compression [1, 14, 21, 25, 28, 30, 34–36]. A complete routine cardiac evaluation (electrocardiography, blood pressure reading, and echocardiography) is mandatory before surgery. The assessment of current hemodynamic stability can give further indications for the need for additional medications or tests [37–39]. Cardiac manifestations may be severe valvular disease (valve thickening and dysfunction dysplasia of the subvalvular apparatus), unstable coronary syndromes, cardiomyopathy, pulmonary hypertension, decompensated heart failure, and significant arrhythmias. Moreover, atrial dilatation, endocarditis, myocarditis, and ventricular aneurysms might be observed [14, 19, 20, 29, 37–40].

### Premedication

Narcotic premedications are to be avoided if airway problems are anticipated, as is usually the case [3, 33]. If a benzodiazepine is administered as premedication, patients should be strictly monitored with a pulse oximeter [1, 40]. The use of available oral drying agents is helpful [40]. Perioperative treatments may include nasal decongestants to control excessive mucus production and steroids to reduce swelling [10, 14, 22, 31].

### Induction of anesthesia

The first consideration is to identify the correct positioning of the patient. Useful information includes a history of obstructive sleep apnea and the child’s favorite sleeping position since this may be the position in which the airway is held open [7]. A small shoulder roll improves airway patency during mask ventilation [5, 27]. Placing the child in a lateral position can avoid airway obstruction by the tongue. Almost all the intravenous and inhaled anesthetics have been described in the studies that are included in the bibliography. Inhalational induction with sevoflurane is sometimes unavoidable to establish reliable venous access. Two-person mask ventilation is often necessary. The use of an upside-down facial mask has been described [3, 33]. The skill lies in being able to keep the airways open and secure them. Ketamine should be the ideal drug [13], but it may increase the amount of secretions. Full apneic doses of narcotics should not be administered before tracheal intubation [33] and it is not advisable to paralyze MPS children before securing the airway [34]. Spontaneous ventilation techniques using oxygen and a high-concentration volatile anesthetic is commonly used. Insertion of an LMA will often improve ventilation [7, 32, 41–45].

### Endotracheal intubation

**Fig. 2:**
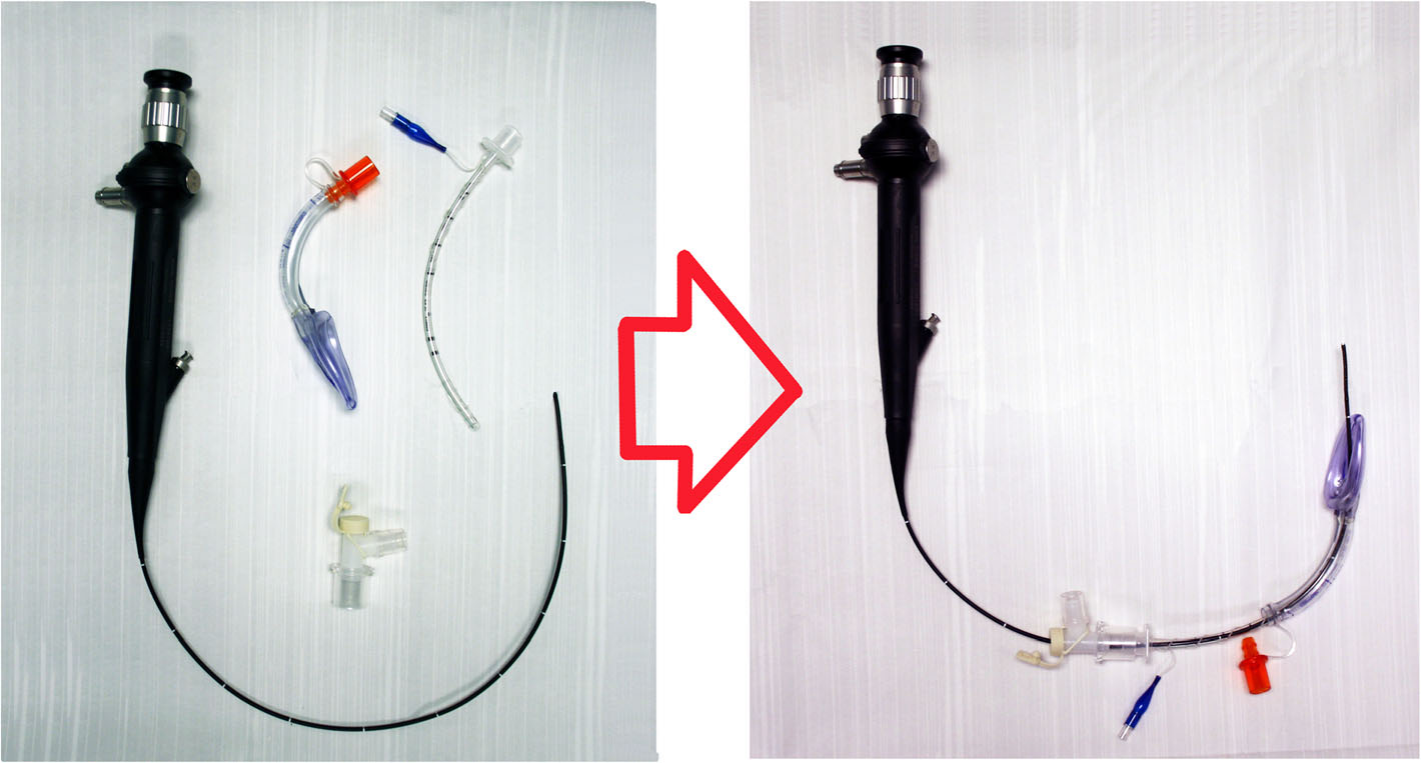
Equipment for FOB intubation through LMA (left) and a method that allows oxygenation and ventilation during the procedure (right)

MPS patients may be very difficult to intubate, regardless of the choice of equipment [27]. The use of videolaryngoscope alone or in combination with FOB has proved to be a useful tool for intubating the trachea [3, 8, 11, 17, 18, 27, 42, 43]. If FOB is not available, displacing the tongue anteriorly by manual retraction helps to access the larynx once the videolaryngoscope blade is inserted. Difficulty with nasal FOB is to be expected because of the narrow nasopharyngeal path and GAG infiltration of the adenoids [4, 9, 20]. Intubation can also be obtained by passing the fiberscope through the LMA [11, 43, 44], and the new supraglottic airway device makes this procedure even easier [46, 47]. The equipment is shown in Fig. 2. The correct size of the endotracheal tube is often smaller than that predicted for the patient’s age to reduce the risk of postoperative subglottic edema [9]. When the patient has a significant risk due to spinal cord compromise from an unstable cervical spine, it is advisable to monitor the somatosensory evoked potentials throughout the perioperative period to assess spinal cord integrity during intubation and positioning maneuvers. Even though manual in-line stabilization of the neck might be sufficient to protect the spinal cord from excessive movements during intubation, there is the risk of increasing the index of difficult intubation [20, 21, 23].

Once general anesthesia is started, the trachea and bronchi can be monitored with a FOB, and a plan for extubation can be made.

### Extubation

Extubation of the trachea at the end of general anesthesia may represent another major risk. A postobstructive pulmonary edema may further worsen the airway patency, resulting in the need for urgent reintubation or tracheostomy [1, 9, 16, 48]. Patients should be extubated when fully awake and after having performed a leak test, and then monitored closely for early signs of upper airway obstruction. However, if at the end of surgery extubation criteria are not present, awakening may be delayed and carried out in the intensive care unit to allow the safe weaning from mechanical ventilation, aggressive chest physiotherapy, and the early detection and treatment of respiratory infectious complications [3, 12, 16, 20, 21, 40]. Preoperative determination of the child’s favorite sleeping position may give some information as to the most appropriate positioning to use as the residual effects of the general anesthetic dissipate [34].

### Tracheostomy

Tracheostomy can be indicated to treat refractory progressive upper airway obstruction or for emergency airway management but, in these patients, placement of the tracheostomy tube can be difficult due to the distortion and to the laxity of the trachea. Tracheostomy may be further associated with stomal narrowing, granulation formation, infrastomal tracheal stenosis, wound infection, and tracheomalacia. For these reasons, the prophylactic use in cases of elective surgery is not routinely recommended, especially in children, and should be discussed in depth with the multidisciplinary team [9, 49–51].

### Anesthesia versus sedation

Radiological evaluations and other tests may require deep sedation in younger or uncooperative patients. Performing deep sedation in remote locations can be a challenge due to the risk of airway obstruction and desaturation. The decision to use deep sedation must be made based on the respiratory conditions of the individual patient; some of them are eligible for deep sedation with native airways [5] while, for others, LMA in spontaneous ventilation or general anesthesia with endotracheal intubation is preferable [12, 52].

### Regional anesthesia

The use of regional or blended anesthesia in MPS patients is still a controversial topic; a careful evaluation of the risk–benefit ratio has yet to be performed. The limited literature consists of case reports of single patients or of small groups of patients. Some successful cases reports are described [34, 53], sometimes associated with deep sedation [54]. Theroux et al., in a retrospective study on MPS IVA children, describe six cases of successful epidural catheters placed for postoperative analgesia. A caudal approach was preferred to a lumbar one in four children because of irregularities of the vertebral bodies and frequent kyphosis [27]. On the other hand, a case of failure is also reported, where the authors hypothesize the deposit of mucopolysaccharides in either the general epidural space or in the sheath of the nerve fibers which prevented the direct access of the local anesthetic to the nerve [55]. Furthermore, Drummond et al. report a case of complete paraplegia in a girl with MPS IV immediately postoperatively after an apparently uneventful lumbar epidural-general anesthesia; the patient sustained a spinal cord infarction, likely due to spinal cord compression or to hypoperfusion. The epidural anesthetic contributed to the delay in the recognition of the paraplegia. The authors concluded that it may be prudent to avoid the use of epidural anesthesia, to support blood pressure in the presence of even moderate spinal stenosis, and to avoid flexion or extension in intraoperative positioning [56].

### Adult patients

With improved care, the life expectancy of patients with MPS continues to increase. They often need surgical intervention for a variety of indications. Thus far, very little literature is available about adult patients, but the increased life expectancy associated with enzyme replacement therapy and hematopoietic stem cell transplantation goes along with an increased demand for surgery and anesthesia. Aging can be associated with severe narrowing of the larynx or trachea and with severe obstructive sleep apnea [26]. The progressive involvement of many organs leads to death. Several cases are reported of successful intubation [36, 46, 50, 57–61] and, in most cases, FOB was used. In one case, a pre-recovery tracheostomy was performed to avoid extubation problems [51]. Cade et al. described a case of MPS VI in which both the supraglottic and subglottic tissues were notably enlarged, and the upper trachea was abnormal. Despite significant facial swelling at the end of the operation, the trachea was extubated without incident [61]. Two papers describe three cases where adults with MPS IVA died of acute respiratory distress syndrome due to systemic storage materials in multiple tissues [62] or to distortion and laxity of the bronchial tree [51].

### The effect of therapy on airway management

Although early diagnosis and therapy give promising results, there is not enough conclusive evidence of the effectiveness of therapies in reducing anesthesiological risks. Two retrospective chart reviews published in 2012 show that enzyme replacement therapy alone does not reduce the incidence of difficult airway management in MPS I, II, or VI, while hematopoietic stem cell transplantation patients have a much lower incidence of airway complications [12, 63]. Another retrospective study reports that enzyme replacement therapy followed by hematopoietic stem cell transplantation does not decrease the overall incidence of difficult airway management related to general anesthesia [42]. A recent prospective study on MPS IVA children who underwent hematopoietic stem cell transplantation shows fewer surgical interventions than for untreated patients, while surgical frequency for patients treated with enzyme replacement therapy was not lower than that of untreated patients [64]. On the other hand, many lines of evidence support the opinion that the treatment of patients with MPS should occur early, at least at the onset of clinical disease, if not presymptomatically [65].

## Conclusions

Patients with MPS have a high incidence of difficult ventilation and endotracheal intubation associated with cardiopulmonary impairment. Spinal involvement poses additional difficulties to anesthesiologists. Any elective surgery requires a preoperative evaluation of anesthesiological risk factors and the availability of a spectrum of airway management equipment. Anesthesia should be performed by a team with expertise in MPS disorders and in the use of advanced airway devices. Literature on MPS largely consists of retrospective case series and case reports, while prospective studies and randomized controlled trials are lacking. Further research with controlled studies on the clinical effect of early therapy, as well as the evaluation of anesthetic risk with the new devices available, would be useful.

## Key messages

- What is already known about the topic: anesthesia for mucopolysaccharidosis patients is associated with high morbidity
- What new information is added by this review: we suggest a procedure for dealing with airway management which could be useful to anesthesiologists who have to take care of these patients

## Additional file

Additional file 1: PRISMA flow diagram. (PDF 77 kb)

## Abbreviations

FOB: Fiberoptic bronchoscope
GAG: Glycosaminoglycan
LMA: Laryngeal mask airway
MPS: Mucopolysaccharidosi(e)s
MRI: Magnetic resonance imaging
