# Supplementary material for: Anesthesiological risks in mucopolysaccharidoses
Source: Ital J Pediatr. 2018 Nov 16;44(Suppl 2):116. doi: 10.1186/s13052-018-0554-1 (PMC6238251; doi:10.1186/s13052-018-0554-1)
Supplement: Supplementary file 1 — PRISMA flow diagram. (PDF 77 kb) [file 13052_2018_554_MOESM1_ESM.pdf]

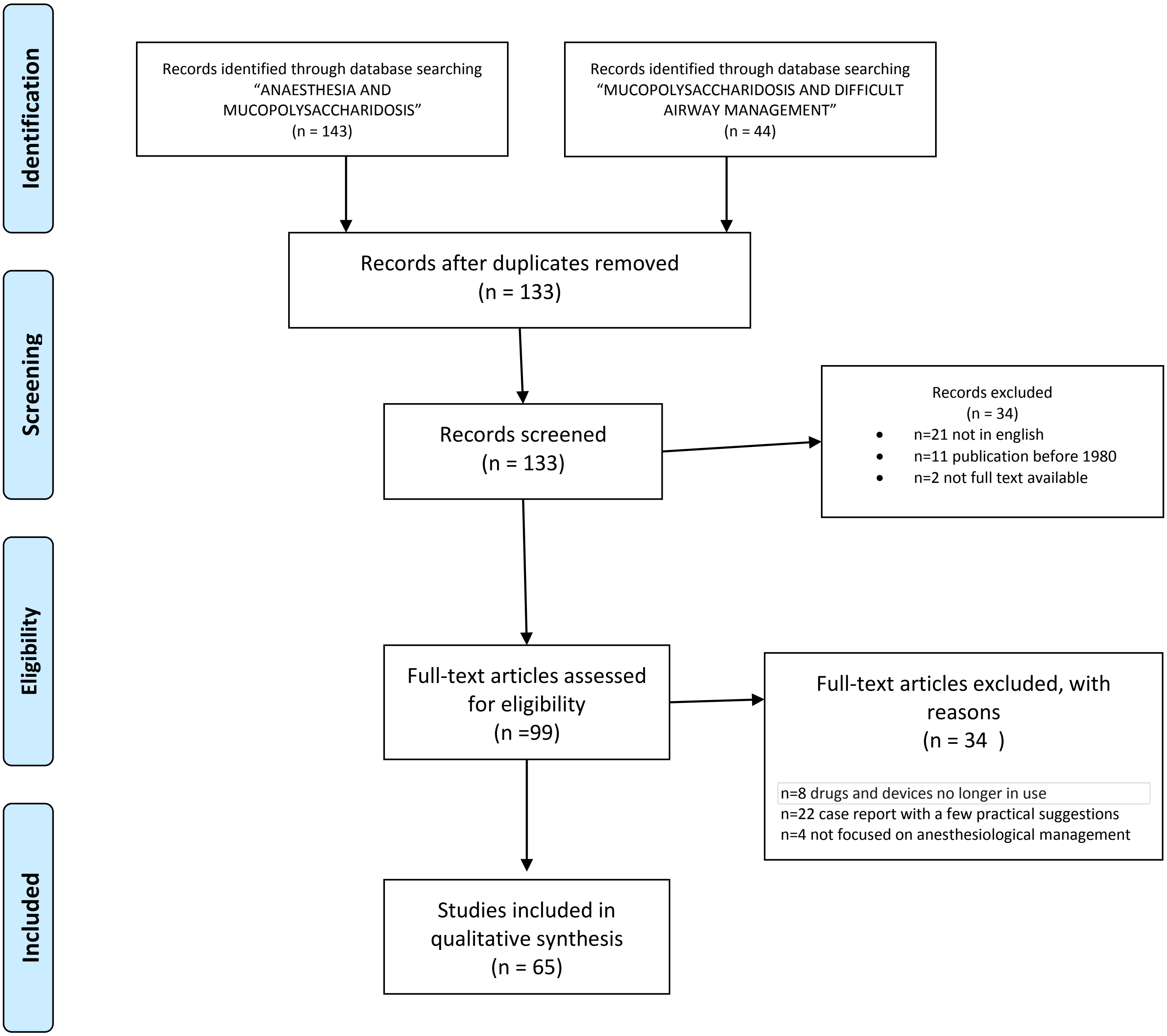

Identification
Screening
Eligibility
Included
Records identified through database searching
“ANAESTHESIA AND
MUCOPOLYSACCHARIDOSIS”
(n = 143)
Records identified through database searching
“MUCOPOLYSACCHARIDOSIS AND DIFFICULT
AIRWAY MANAGEMENT”
(n = 44)
Records after duplicates removed
(n = 133)
Records screened
(n = 133)
Records excluded
(n = 34)
n=21 not in english
n=11 publication before 1980
n=2 not full text available
Full-text articles assessed
for eligibility
(n =99)
Full-text articles excluded, with
reasons
(n = 34)
n=8 drugs and devices no longer in use
n=22 case report with a few practical suggestions
n=4 not focused on anesthesiological management
Studies included in
qualitative synthesis
(n = 65)
